# Impact of the global chip shortage on the development of in-memory chips

**DOI:** 10.1038/s41467-022-31598-5

**Published:** 2022-07-15

## Abstract

Lockdowns due to the pandemic in the last two years forced a critical number of chip-making facilities across the world to shut down, giving rise to the chip shortage issues. Prof. Meng-Fan (Marvin) Chang (National Tsing Hua University, TSMC—Taiwan), Prof. Huaqiang Wu (Tsinghua University—China), Dr. Elisa Vianello (CEA-Leti—France), Dr. Sang Joon Kim (Samsung Electronics—South Korea) and Dr. Mirko Prezioso (Mentium Techn.—US) talked to *Nature Communications* to better understand whether and to what extent this crisis has impacted the development of in-memory/neuromorphic chips, an emerging technology for future computing.

Please tell us about your background, your research and its motivation driving the development of in-memory/neuromorphic chip.

**MC:** Since I completed my training in electrical engineering, I have spent more than 12 years working in industry and 13 years in academia. I am currently serving as a professor at the National Tsing Hua University (NTHU) in Taiwan. Over the past two decades, I have developed a range of commercial products and involved with the research of devices, circuits, and architecture of memory. My research interests include memory circuit design, ultra-low-power systems, 3D-IC, circuit-device interactions, circuits for neuromorphic computing, and in-memory-compute for artificial intelligence. For quite some time, I have been particularly interested in methods aimed at overcoming the memory wall issue in conventional computing architectures. In-memory-compute and neuromorphic chip have become promising revolutionary methods to enable significant improvement in performance and energy efficiency for computing. My experience in industry and academia has taught me that impactful research requires a balance between novelty and practical realization. Therefore, my research on in-memory/neuromorphic chip spans multiple disciplines from devices to circuit design and even the formulation of architectures and algorithms, with consideration of manufacturability.

**Figure:**
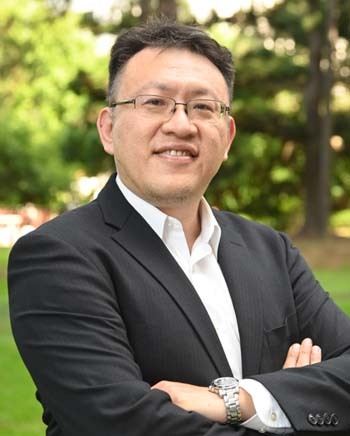
Prof. Meng-Fan (Marvin) Chang

**HW:** I was initially trained as a material scientist but later on my research work expanded more into emerging memories and neuromorphic chips. I am currently a professor in the School of Integrated Circuits at Tsinghua University. As artificial intelligence (AI) demands for higher and higher computing power and energy efficiency, conventional silicon chips face the so-called von Neumann bottleneck amid the slowdown of Moore’s law scaling. In searching for the solution to next-generation computing chips, we were fascinated by the extreme energy efficiency of human brain, which led us to work on neuromorphic computing using emerging nonvolatile memories as artificial synapses, in particular RRAM (or memristor) with analog resistive switching characteristics. The key motivation here is that we can implement the in-memory computing architecture on a RRAM crossbar array, where the vector-matrix multiplication, the most computation-intensive operation in artificial neural networks, can be done in a single step via fundamental physical laws. This is an extremely appealing computing paradigm that can break the von Neumann bottleneck to achieve orders of magnitudes higher energy efficiency than CPU and GPU.

**Figure:**
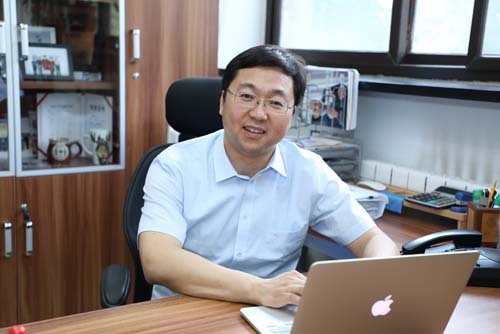
Prof. Huaqiang Wu

**EV:** My background is in electronic engineering. Since the beginning of my career, I have worked in the field of non-volatile memory devices. The leap towards cross-disciplinary research came when I was offered a staff scientist position at CEA-Leti institute Grenoble (France) to work on developing new applications for emerging resistive memory devices (also called memristors). Once I started working on memristors, I realized almost immediately that there was a great new opportunity for this technology if we could implement novel functions exploiting their physics, instead of using them only as conventional digital elements to store one bit of information. This new approach to using memories differs from the way today’s conventional computers operate. Indeed, conventional computer simulations disregard the underlying physics of the hardware they run on. Exploiting the physics of memristors to create new functions is similar to the way the brain works. In the brain, some of the computing takes place within the network of biological memories (or synapses), thus avoiding data movement between the processor and the storage unit and we know that moving data is the main reason for the high energy consumption in machine learning applications running on conventional computers. My current project is to take inspiration from insects’ nervous systems to relax hardware requirements in terms of memory density and reliability, and to build energy-efficient, uncertainty-aware, and adaptable learning and inference engines. I am convinced that a holistic research approach is essential to develop low-power architectures inspired by the human brain and many other tiny animals, where process development and integration, circuit design, system architecture and algorithms are simultaneously optimized.

**Figure:**
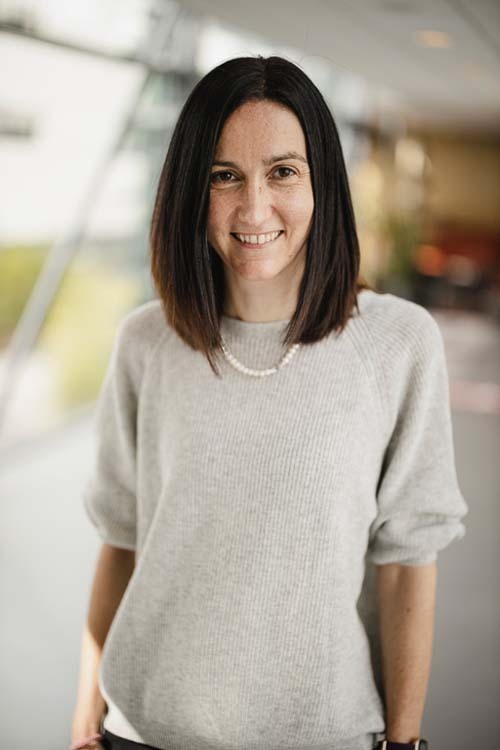
Dr. Elisa Vianello

**SJK:** After graduating from college, I started my career as an electrical engineer by working at a powerline communication startup. At that time, my main role was to develop wireless communication algorithms in the self-developed chip. Afterwards, I did research on information theory in the doctoral program at Harvard University. My research was mainly focused on theoretical analysis of information exchange between multiple objects sharing communication media. After completing my Ph.D degree, I joined Samsung Advanced Institute of Technology (SAIT) and have conducted various projects related to system level architecture. I have developed wireless power and data transmission, ultra low-power system for mobile healthcare applications, and implantable system for electroceutical. These tasks were mainly cross-optimization researches from system to circuit design. In particular, while studying bioelectronics, I became interested in brain inspired systems, and eventually started researching in the in-memory/neuromorphic field towards the next generation AI technology.

**Figure:**
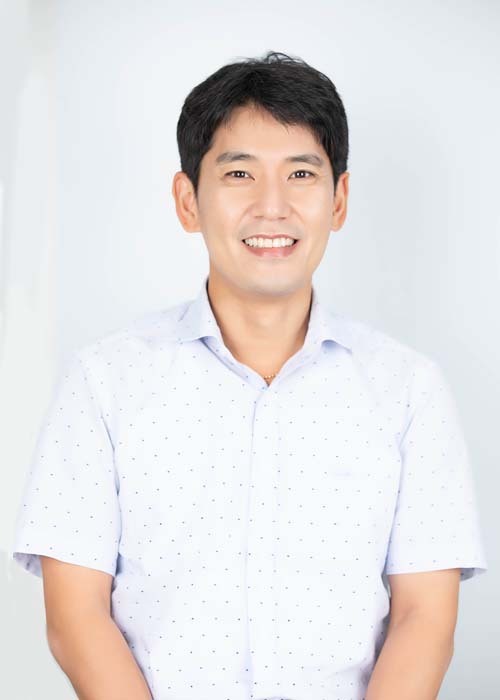
Dr. Sang Joon Kim

**MP:** My academic formation starts as a condensed matter physicist. I was working on magnetic and resistive memory devices in the CNR institute in Italy when I came to know the seminal work of Prof. Dmitri Strukov in the memristor field. At that time, he had an open position at the University of California Santa Barbara, and after meeting with him, I moved to California, to join his lab. There, my interest, expanded from memory device dynamics to Neural Networks and Neuromorphic applications, a fascinating field with very high potential impact. The teamwork in the lab was very successful and led to great results. These results were the motivation that drove us to bring that technology to the market. Of course, during these years, we have done a lot development and what we have now is different from what we had at that time, but it is definitely an evolution started by that initial seed.

**Figure:**
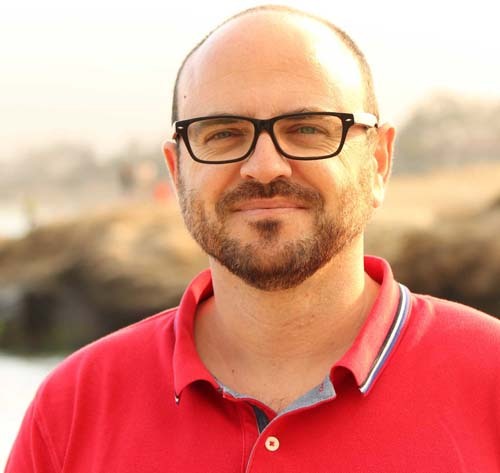
Dr. Mirko Prezioso

How far do you think your research is away from the commercialization of in-memory/neuromorphic chips? Any major bottleneck?

**MC:** Numerous research results have demonstrated that in-memory-compute and neuromorphic chips can outperform conventional chip designs based on the von Neumann architecture. Moreover, with regards to in-memory-compute methods, many companies have been assessing whether it would be feasible to manufacture and implement this technology for commercial purposes. The major challenges in implementing new products based on in-memory-compute schemes include (1) a tradeoff between gains in PPAC (power, performance, area, cost) versus the added costs associated with designing and verifying novel architectures, circuit operations, chip integration, and algorithms, (2) flexibility in the application of new technologies to wide range of applications, and (3) scalability and portability across product generations and manufacturing process technologies (technology nodes, such as 22 nm, 16 nm and 5 nm). The major challenges in manufacturability include (1) integrating devices and circuits within the manufacturing constraints stipulated by semiconductor companies, (2) achieving consistent accuracy over a range of operational voltages and temperatures, and (3) achieving mass production while maintaining high yield with high reliability and low cost. A number of leading companies have recently set aside their initial skepticism of in-memory-compute, and some have actually included these developments in their product roadmaps. A number of our partners in industry have even begun developing in-memory-compute solutions based on technology transfers that began with our research. We are aware of that a number of commercial chips using in-memory-compute methods are expected to appear on the market in the near future.

**HW:** We, along with many colleagues around the globe, have been working very hard to commercialize in-memory computing technology in the past decade. At Tsinghua, we have worked closely with leading foundries and developed multiple platforms, from 130 nm to 28/22 nm, to integrate RRAM with advanced Si CMOS for building large-scale in-memory computing chips. It is also encouraging to see that major chip makers, including TSMC and Intel, are also getting into this business. We hope to have some in-memory computing chips available on the market in about 3–5 years, which can run various large-scale neural networks like ResNET and tackle real-world AI problems efficiently. While prototype RRAM-based in-memory computing chips have already shown promising computing power and energy efficiency, and also enormous potential in applications like edge computing, there are certainly challenges ahead. For example, the variability and reliability of RRAM devices need to be carefully addressed through material engineering and process optimization. To further boost the computing power of in-memory computing chips, it is also challenging to increase the number of RRAM devices integrated on to Gb level and above, for which more advanced technology nodes and integration techniques are required. Finally, to build large-scale chips based on an emerging device and a new architecture, a system-to-technology co-design methodology is highly favored, for which accurate device models and related electronic design automation (EDA) tools need to be developed.

**EV:** The energy efficiency of current commercial neural network accelerators peaks at several dozen tera-operations per second per watt (TOPS/W), which is about six orders of magnitude below the efficiency of the human brain. That is the main reason why taking inspiration from biology is a good idea. The neuromorphic engineering academic community is mostly focused on the development of small-scale (analog or mixed-signal) prototypes with the dual objective of understanding neural computation and developing sensory processing architectures. Thanks to the advances in CMOS-VLSI technology, neuromorphic digital chips could integrate very large numbers of silicon neurons, while keeping the power consumption extremely low. My work on non-volatile memories falls into these two new areas. On-chip non-volatile memory capacity is the main factor limiting significant reductions in the energy consumption and the execution time of today’s neuromorphic digital inference hardware. Microelectronic foundries already started risk production of resistive memories using advanced technological node process. The next step will entail demonstrating how to mass-produce them. Being capable of programming resistive memories to intermediate states between their lower and higher resistance values is essential, if we want to store the synaptic weights of ultra-low power neural network analog circuits. This compact storage method could be used in a wide range of applications, such as Bayesian, spiking and artificial neural networks. However, in addition to a multi-level capability, the distinct applications demand different hardware specifications. For example, the latest state-of-the-art spiking neural network models require memory elements operating at multiple timescales (from milliseconds to hours). In analog neuromorphic hardware, unlike digital circuits, we do not have the option to store intermediate data until needed. This is why, in order to implement multiple timescales, we need resistive memory devices with both non-volatile and volatile properties. The ideal multi-level memory with tunable volatility does not exist today. A broad range of materials, devices, and techniques are still being actively investigated to find the ideal memory (synapse). In parallel, tremendous progress has been made in computational neuroscience and machine learning. This is the best time to combine the know-how gained so far in all these disciplines to obtain breakthroughs that can potentially solve many of the problems that information and communications technologies (ICTs) are starting to face.

**SJK:** In my research, I am seriously considering commercialization as well as development of innovative technologies which will overcome current limitations. In fact, my research spans circuit design, system architecture, and AI algorithms. Specifically, I am developing custom systems in our company’s fab that can be immediately applied to business after verification of the technology. For a technology to be commercialized, it is generally accepted that at least one aspect must be superior to the existing technology in terms of PPAC (Power, Performance, Area, Cost), while at the same time all other aspects must not be inferior. In-memory/neuromorphic technology has a clear advantage over conventional digital computation in terms of low-power computation (power). However, the overall performance degradation due to analog operation and the area overhead due to combining arithmetic functions with memory are technically critical issues that need to be overcome. Additional process cost for producing such memory should be considered as well.

**MP:** We are not far from production; we are on course for the end of 2023. There are no fundamental bottlenecks, our technology and architecture has been proven on Silicon, and we are executing the engineering toward the production.

In view of the current global chip shortage caused by pandemic, do you think this crisis has an impact on the overall sector of in-memory/neuromorphic chips?

**MC:** The global chip shortage has certainly had a profound impact on research and development pertaining to in revolutionary methods like in-memory-compute and neuromorphic chips. Given these difficult operating conditions, many companies have opted to assign their secured wafer volume to the mature (i.e., risk-free) high-volume products. Indeed, it is important to consider that semiconductor companies prioritize high-volume product-type chips over research projects in the assignment of multi-project wafer (MPW) shuttle services and full-mask wafer runs.

Most of the products that utilize in-memory-compute are still in the prototype development or trial-run stage, and many of the revolutionary techniques developed for in-memory-compute and neuromorphic chips are still in the early stages of research.

So, throughout the chip-shortage crisis, many university researchers and start-up companies have had difficulty securing resources for chip fabrication (e.g., silicon area and access of multi-project wafer shuttle for testchips). University teams have also had to face an uncertain future in terms of tapeout opportunities, technology node availability, and wafer output dates.

**HW:** Yes, the current global chip shortage crisis hurts almost every chip product, and it certainly has a big impact on the in-memory computing chips, especially in terms of chip tapeout (i.e., chip fabrication in a foundry). If you read the news, most foundries are running out of capacity for mature products, such as automobile electronics, microcontroller units (MCUs), and display drivers, etc., so their investments on developing new technologies may be affected. As a result, it has been quite challenging to find a place to fabricate in-memory computing chips, which are still in their development phase, and sometimes the tapeout schedule is pushed back by months or even longer. Besides manufacturing, the chip packaging and testing has also been held back due to the current crisis. We hope the situation could gradually get better in the next year or so as foundries are ramping up their capacities as well.

**EV:** The global chip shortage currently affects all types of components, including digital processors based on advanced technology nodes and analog chips based on older technology nodes. It will certainly affect the implementation of approaches that use standard digital computation techniques, due to the difficulty in accessing standard FPGA or CPU/GPU/MCU. However, specific in-memory/neuromorphic chips with dedicated technology solutions (i.e., resistive memories) are still in development in academic and research laboratories in collaboration with the Integrated Device Manufacturers (such as Intel or STMicroelectronics) and Foundries (such as TSMC and GF) and therefore they suffer less from market fluctuations. I hope that the standard chip shortage will progressively be solved in the coming year to 18 months and that by that time the exciting new in-memory/neuromorphic chips we are developing will have gained larger acceptance and will be on their way to be incorporated into an increasing number of computational tools.

**SJK:** I believe the global chip shortage has limited impact on in-memory/neuromorphic research. In-memory/neuromorphic research is in a relatively early stage in terms of commercialization, and there still remain many technical issues which need to be overcome. For commercialization, it is necessary to comprehensively consider not only device and chip development, but also AI algorithm, software framework, as well as hardware system architecture. Being such a multidisciplinary research, I don’t think it would be directly affected by the supply chain shortage issue.

**MP:** In short, no I do not think there will be major effects. In more detail, this is quite a complex question that implies some kind of forecast for the future of chip production. A very complicated forecast to make, as the semiconductor technology is, nowadays, equivalent to oil, in terms of strategic importance and intertwining with the geopolitical situation. Moreover, the semiconductor production chain extends on a global level, with no single country nor continent owning it completely. This means that there are many interests and factors at play in a complex network. Nevertheless, a first simplification can be done by diversifying between mature technology nodes and lead nodes. The first are those chip production methods that are older, cheaper, and well established (able to deliver features down to 22 nm in size). The latter, the lead nodes, are represented by the cutting edge of the processing technology, able to condense the highest number of transistors per area as if the feature size were from 14 nm down to 3 nm. The lead node production sees TSMC as the main producer followed by Samsung. The 7 nm and 5 nm are very tight on production capacity as many big corporations utilize these nodes for high-volume products. However, I am sure TSMC will be able to deliver in these nodes, especially as their new 3 nm gets into mass production in the second half of this year. On the other hand, the mature nodes’ production capacity is not as tight as is the case for the lead ones. Therefore, in the short period, I think those smaller companies that are relying on lead nodes for production could see some setbacks at the production phase, while those that do not need to push on the scaling factor, i.e., the reduction in feature size by moving to the lead nodes, will not see such a problem.

How is the chip-shortage crisis impacting on your research roadmap? Any adjustment to cope with it?

**MC:** A lack of resources and an uncertain opportunity for such research-type chip fabrication has had a profound effect on our research roadmap. We have sought to overcome these limitations by expanding the scope of our research beyond macro-level in-memory-compute circuit design to include developments at the system and software levels. We have also adopted the software-system-circuit co-design methodology, adjusted our research focus, and sought to minimize the silicon area required to prove our design concepts and demonstrate our testchips. Our team has had to adjust our testchip design schedule and book the MPW shuttle for chip fabrication at least a few months earlier than our usual schedule. We have also been forced to increase the man-power required for porting testchip designs to another alternative manufacturing process technology as a back-up plan by which to take advantage of multi-project wafer shuttle space when it becomes available at the last minute. Essentially, the chip-shortage has forced us to adjust the scope of our research and use our resources more efficiently.

**HW:** As mentioned above, the chip-shortage crisis has also impacted our in-memory computing research especially by delaying chip tapeout. It usually takes multiple chip iterations to go from academic research prototypes into a viable technology for commercialization. Even before this chip-shortage crisis, we started working closely with foundries to develop advanced RRAM technology for in-memory computing applications. By convincing the foundries that this is a truly promising and valuable technology, we are getting enormous supports from them, especially on the process development and chip tapeout. Through these collaborative efforts, we hope to reduce the impact of chip-shortage crisis on our research roadmap to the lowest level. In the meanwhile, we have been working on testing existing RRAM chips and building an end-to-end simulator for in-memory computing, which is fully calibrated by experimental results from our hardware. We believe such simulator could provide an extremely valuable guidance for designing and validating large-scale chips in the future. It is also helpful when communicating with users and industry.

**EV:** In our case, the fact that we execute a large part of the process ourselves and have long-term partnerships with chip manufacturers to obtain the necessary base wafers, means that we are not really affected. More generally, the concern for the roadmaps comes from the barriers that we see emerging across the world regions. Both the current climate of geopolitical uncertainty and the travel restrictions following the global health crisis are negatively affecting the scientific exchanges and slowing down the diffusion of new ideas across the scientific community in the pre-competitive phase.

**SJK:** The impact of the chip shortage crisis on my research progress so far is not significant. SAIT (Samsung Advanced Institute of Technology) is researching future technologies that Samsung electronics aims to commercialize. The core technology completed in SAIT is transferred to relevant internal business departments and additional development is conducted for commercialization. As a part of that, current in-memory/neuromorphic research is being conducted mainly in SAIT. I have been communicating with related business department while using our company’s MPW (Multi Project Wafer) service for research level chip development. So far there have been no schedule delay issues due to chip shortage.

**MP:** There has been no major impact on our research roadmap due to the chip shortage. Our prototyping and pre-production volumes are not an issue from the production point of view. Moreover, linking to what I said before we do not rely on scaling to achieve the performances we deliver, but rather on the architecture so we do not have to rely on lead nodes. However, the chip shortage is not only a matter of production but of distribution as well. The logistic network has been disrupted for all the reasons we know (in brief: COVID-19 increased product demand while reducing the workforce and the product bandwidth in logistics hubs) and that can have an impact in the short term (1-2 years) for everyone. Possibly from a cost perspective rather than purely from an availability point of view. This could be a factor in the semiconductor economy in general, and we could see a downturn, a slow-down of the semiconductor financial market, in the incoming months. However, this is hardly a big issue, as it would be the 14^th^ downturn in a market that showed very high resilience and always came back better and stronger than before. I am quite optimistic for the longer term, given the incredible increase in investments from the semiconductor producers to expand and distribute the production facilities, together with a distribution chain that, even earlier, will adapt to the current difficulties.

Any suggestions on how to incentivize researchers, inventors, industrial partners, investors or even policy makers to navigate through the crisis by a collaborative effort?

**MC:** Obtaining sufficient chip fabrication resources from industry is essential to maintaining momentum in fundamental and application-oriented research of in-memory-compute and neuromorphic chips during the chip-shortage crisis. This can be achieved if all members of the research and development (R&D) ecosystem collaborate closely to improving the high-level value proposition and the future manufacturability of in-memory-compute and neuromorphic chips from industry perspective. Below, I list my suggestions pertaining to the R&D ecosystem. Researchers need to interact closely with partners in industry in order to understand the challenges of transforming fundamental research results into viable commercial products. Researchers must also work to minimize the gap between fundamental research and product-oriented prototypes. Inventors need to enhance the communication with researchers, in-house production teams, external industrial partners, and even other investors to balance novelty and performance gain against feasibility for maximizing the value proposition and proving the manufacturability of their product-oriented prototypes. Policy makers should negotiate with semiconductor integrated-device-manufacturer (IDM) and foundry companies to secure chip fabrication resources for academic research. Policy makers should also work toward increasing funding and evaluation systems to award research on cross-layer integration (e.g., devices, circuits and systems) for narrowing the gap between fundamental research and the development of commercial products.

**HW:** For a new and yet promising technology like in-memory computing, we would definitely need more than just one or a few research groups on the way to commercialize this technology. On the research side, open-source hardware platforms shall be established and offered to a broader community, so that we can attract researchers and inventors worldwide across different disciplines and facilitate collaborations. This is something we have been working on lately at Tsinghua. A model example could be the IBM Quantum Experience, which offers cloud access to one of the most advanced quantum computers available. On the funding and policy side, more investments from both industry and governments are definitely needed to continuously support and accelerate the developments of in-memory computing chips. This is especially important during this chip-shortage crisis by the pandemic while the chip tapeout cost increases dramatically.

**EV:** There are multiple national and supranational possible incentives. At the European level, the Chips Act was recently launched by Ursula Von der Leyen and Thierry Breton to boost competitiveness of European microelectronics for the next 10 years. In the European Chips Act, opportunities to encourage collaborative research are clearly stated, in particular in pillar 1 ‘Chips for Europe Initiative’. Note that a similar initiative has also been launched in the US. These EU and US initiatives encourage collaborations between research centers and industrial players in order to establish a continuum of activities in a lab-to-fab model. At the national level, the France2030 initiative was launched by President Emmanuel Macron; supporting microelectronics research activities is one of its main goals. I believe more opportunities of this kind should be offered, involving teams scattered in several countries, to support cross-fertilization among foundries, chip designers, end users and academia. Working together is fundamental to assess how in-memory computing/neuromorphic chips based on novel microelectronics technologies (i.e., embedded resistive memory devices) are meeting and will meet the real needs of the market today and in the future.

**SJK:** In general, it is a difficult situation and honestly, I do not have an answer to it. For the in-memory/neuromorphic research field, it requires close interdisciplinary collaboration between various research fields. In terms of chip development, current shortage crisis may have some impact, but I think it is more important to solve many technical issues through collaboration for commercialization. When it comes to in-memory computing, there is a clear limit to solve analog computation errors and area overhead by circuit design alone. It is necessary to think together from an algorithmic and system point of view. Neuromorphic research should build close collaboration including bioelectronics and brain research fields and show clear advantages over current AI algorithms.

**MP:** One major factor that will drive the future of the semiconductor economy is the competition, or better yet the technology war, between China and Western countries. It is a great danger that will require doubling the efforts and costs to achieve a slower technology development. We should find a way to collaborate, and this means we should all respect each other and play by the same rules. Other than that, I think investments are the key factor in technology businesses. Every regional area has different policies and business environments of course. The US has a great Venture investment network, both institutional and private. Yet, investments in hardware are always more difficult to be found than software, even in the US. As for Europe, I think there should be much more investment in general, more opportunities for startups and in particular, of course, those focused on hardware technology. I know many great people there helping in that direction and I can see the trend is there. Hopefully, the war at Europe’s door will soon be over so that not only we will all be relieved by its horrors, but then the economy will be able to give us all more opportunities.

*This interview was conducted by Nature Communications editors Dr. Selina La Barbera and Dr. Congcong Huang.*

